# Interconnection between pastures, grazing ecosystem, animal welfare, meat quality, and human health

**DOI:** 10.1093/af/vfaf041

**Published:** 2025-10-14

**Authors:** María Sol Villaverde, Mariano Menghini, Marcela F Martínez, Nicolás DiLorenzo, Rodrigo D Bravo, Hugo M Arelovich

**Affiliations:** Departamento de Agronomía, Universidad Nacional del Sur (UNS), Bahía Blanca, Argentina; Consejo Nacional de Investigaciones Científicas y Técnicas (CONICET), Godoy Cruz 2290, Ciudad Autónoma de Buenos Aires, Argentina; Laboratorio de Ecosistemas Naturales y Agropecuarios (LENA-CIC), Bahía Blanca, Argentina; Departamento de Agronomía, Universidad Nacional del Sur (UNS), Bahía Blanca, Argentina; Laboratorio de Ecosistemas Naturales y Agropecuarios (LENA-CIC), Bahía Blanca, Argentina; Departamento de Agronomía, Universidad Nacional del Sur (UNS), Bahía Blanca, Argentina; Laboratorio de Ecosistemas Naturales y Agropecuarios (LENA-CIC), Bahía Blanca, Argentina; North Florida Research and Education Center, University of Florida, Marianna, FL 32446, USA; Departamento de Agronomía, Universidad Nacional del Sur (UNS), Bahía Blanca, Argentina; Laboratorio de Ecosistemas Naturales y Agropecuarios (LENA-CIC), Bahía Blanca, Argentina; Departamento de Agronomía, Universidad Nacional del Sur (UNS), Bahía Blanca, Argentina; Laboratorio de Ecosistemas Naturales y Agropecuarios (LENA-CIC), Bahía Blanca, Argentina

**Keywords:** food quality, grass-legume mixed pastures, one health, ruminants, soil fertility, sustainability

**Short Implications**

- Pastoral ecosystems are essential for sustainable livestock production: enhance soil fertility, biodiversity, and carbon sequestration. Legumes interseeded in pastures improve yield and may help mitigate methane emissions.
- Animal health and welfare depend on the production environment, with diet shaping rumen microbiota and influencing digestion.
- Grain-fed systems optimize animal efficiency while grass-fed systems enhance oxidative status and welfare. Consumers may perceive pasture-raised meat as more natural and ethical.
- Grazing animals can accumulate harmful compounds like dioxins from soil and plant contamination, posing long-term human health risks.
- Pasture-fed meat typically has a healthier fatty acid profile, with greater polyunsaturated fatty acids and conjugated linoleic acid concentrations. Grazing also increases antioxidant compounds in meat, though composition varies by species and feeding system.

## Introduction

**Figure 1.**
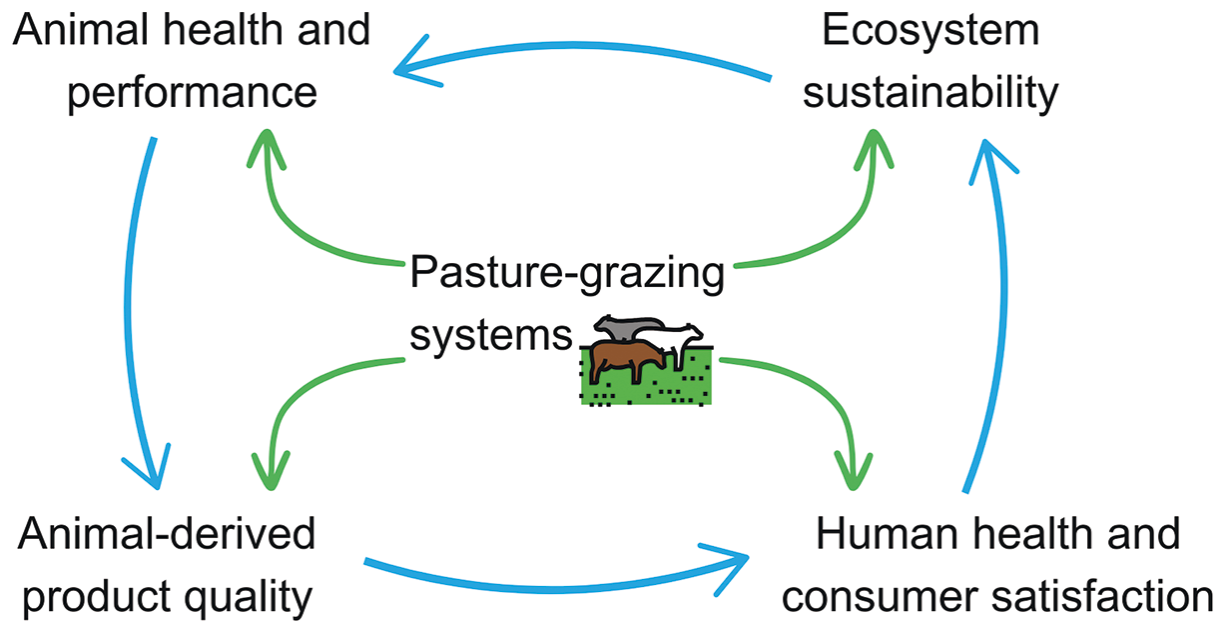
Diagram showing model of interactions between pasture-grazing systems, ecosystem sustainability, animal health, animal-derived product quality, human health and consumer satisfaction. Note the cyclic nature of these relationships and the positive feedback loop.

One health (OH) is an integrated, unifying approach that aims to sustainably balance and optimize the health of people, animals, and ecosystems. The relevance of this concept was substantially expanded when November 3^rd^ was established as the global “OH Day” in 2016. Since then, by the end of 2023, at least 950 events on this topic have been internationally registered (One Health Commission, 2025). The focus of this review is on the interactions among ruminants grazing on pastoral ecosystems, the most relevant impacts on the environment, animal health and welfare, and human health as consumers of the derived animal products. These interactions are schematized in Figure 1.

## Environmental Health of Pastoral-Ecosystems

Rangelands and improved pastures represent 22% of the world’s land surface and approximately 70% of the global agricultural land. However, about 80% of the agricultural land worldwide is used for livestock production, mostly grazing land, with a smaller portion used for fodder production. The remaining 16% is used for food crops, and 4% for non-food crops (Ritchie and Roser, 2019).

Rangelands are composed of diverse native plant communities, whereas sown pastures are typically dominated by one or a few introduced species. Rangelands generally experience less human intervention than seeded pastures, leading to distinct grazing management practices (Török et al., 2024). However, for the purposes of this review, both native and seeded pasturelands will be considered “pastoral ecosystem” when intended only for grazing purposes. *Soil and pastures.*

Like any extractive activity, grazing generates a negative balance of soil nutrients, and organic matter decreases if these nutrients are not returned to the soil. This loss of nutrients could be exacerbated by the export of harvested products (Craswell et al., 2004). Remarkably, due to nutrient recycling, the total mineral extraction per kg of animal body weight is significantly less than plant products like soybeans, wheat, or corn. Consequently, animal production generates greater economic returns per kilogram of mineral exported, adding value to primary pasture production (Craswell et al., 2004).

It is critical to preserve soil fertility and stability for a healthy agroecosystem. According to Lemaire et al. (2023), a level of soil fertility compatible with global food demand could be achieved through the efficient recycling of mineral nutrients within the ecosystem, the food system, and the waste management system. This can be done through natural inputs, which limit losses and keep the soil nutrient balance close to neutral. Thus, if properly managed, pastoral-livestock farming, in addition to contributing to the supply of high-biological-value foods, could offset nutrient depletion through a more uniform distribution of animal waste inputs.

Studies on the short- and long-term impacts of grazing on pastoral ecosystems often show contradictory results due to the complex interaction of the many factors and differences in grazing management strategies. At least three major factors associated with the impact of grazing on the pastoral ecosystem are identified: livestock type, grazing intensity, and pasture type, which are further affected by soil nutrient availability, moisture, and pH (Török et al., 2024). Improper or excessively high stocking rates result in overgrazing and trampling, causing vegetation deterioration, reduced soil water infiltration capacity, biodiversity loss, and decreased livestock productivity. Overgrazing affects approximately 262 million hectares worldwide (Lyons et al., 2023).

Converting cropland to permanent pasture with legumes and perennial grasses, combined with well-managed grazing systems, could significantly benefit ecosystem health and mitigate climate change (Viglizzo et al., 2019). As discussed by Menghini et al. (2024), different legumes increase soil nitrogen (N) through biological fixation, which can replace synthetic fertilizers and enhance plant biomass production (Table 1). This increased biomass enhances soil organic C through decaying plant material and roots. Additionally, forage legumes in grazing systems can return similar amounts of N through plant litter as grasses fertilized with moderate N levels (Jaramillo et al., 2021). Lyons et al. (2023) found that well-managed grasslands can function as carbon sinks as effectively as forests, especially through belowground biomass. In South America, Viglizzo et al. (2019) reported that grazing lands generate carbon surpluses that could not only offset rural emissions but could also partially or totally offset the emissions from non-rural sectors.

**Table 1.** Herbage mass, nitrogen (N) yield, and apparent N fixation in spring in different pastures mixtures in a temperate region in Argentina^†^

*Item*	Perennial pastures		
	Grass monoculture (*Thinopyrum ponticum*)	Grass-legume (*Vicia villosa*)	Grass-legume (*Melilotus albus*)
Herbage mass yield, kg/ha	2,887	4,351	4,177
Legume proportion, %	0	41	31
N yield, kg/ha N	38.4	95.8	72.2
N fixation by legume, kg/ha N	0	57.5	33.8
Urea equivalent^*^, kg/ha urea	0	239	141

^*^Considering a 50% efficiency in the use of agricultural granulated urea 46% N.

^†^ Menghini et al., 2024.

### Gas emissions to the environment

The livestock sector contributes 5% of total anthropogenic greenhouse gas (GHG) emissions, with enteric methane (CH₄) from ruminants accounting for 39.1% (Lileikis et al., 2023). Certain legumes, such as alfalfa, vetch, sainfoin, and birdsfoot trefoil, contain greater concentrations of condensed tannins and saponins, which may help mitigate the ruminal methanogenesis (Vasta et al., 2019). Condensed tannins are toxic to H_2_-producing bacteria and protozoa, and bind to archaea, inhibiting the formation of the methanogen-protozoan complex. Saponins disrupt the cell walls of protozoa and kill them, thereby reducing H_2_ production. However, the reduction in CH₄ production is often associated with lower organic matter digestibility, which may decrease animal productivity (Vasta et al., 2019). Additionally, condensed tannins can reduce ammonia-N production in the rumen by binding to proteins and polysaccharides, increasing fecal-N excretion, which is more stable and produces less N_2_O emissions than urinary N (Orzuna-Orzuna et al., 2021).

Grain-based diets can also lower CH_4_ production by reducing the population of cellulolytic bacteria, protozoa, and methanogenic archaea due to lower ruminal pH. However, this reduction could be offset by GHG emissions from the cultivation of cereals for animal feed, due to the use of herbicides, pesticides, fertilizers, and fuel for agricultural machinery (Lileikis et al., 2023).

## Animal Health and Welfare

Animal health, welfare, and performance are closely linked to their production environment, including feeding strategies, management, climate, soil, and animal type. In pastoral systems, animals obtain food by their grazing abilities, while confined animals receive a complete mixed diet, mainly based on grains in a feeder within fenced pens. Although ruminants can digest grain, this is an entirely different digestion process from that occurring with grazed forages (Parsons et al., 2025). Transitioning from pasture to feed yards requires an adaptation process to the new diet, environment, and limited movement due to reduced available area.

### Ruminant digestive system health

A discontinuous feed input, regulated by the feeding patterns and diet composition, determines the rumen environment conditions. Factors such as solid content, temperature, pH, osmolality, fermentation products, and ammonia-N also influence the ruminal environment as summarized by Wang et al. (2019). The normal ruminal pH range varies between 6.2 and 7.0, but with grain-fed diets, pH could decrease below 5.5 during subacute or acute acidosis events (Commun et al., 2009). This condition affects rumen health, impacting voluntary intake and overall animal performance. This disorder is not expected to occur when animals graze in pastures, although some other problems, like bloat, may appear, particularly when grazing fresh legumes and small grain forages. The latter can be more easily offset with proper grazing strategies.

The rumen microbiota is essential for animal health by sustaining the stability of the rumen environment and its fermentation patterns (Wang et al., 2019). The digestive system of ruminants is particularly linked to the surrounding environment since the microorganisms in their forestomach are the same taxonomic groups found in soil, plants, and even humans (Banerjee and van der Heijden, 2023). Therefore, the relative proportions of each taxonomic group of microorganisms would depend on the environment in which ruminants are maintained. Metagenomic analyses proved that substantial differences can be found in the composition of the rumen microbiota and their resultant metabolites when animals were fed either a grass or a grain diet (Wang et al., 2019).

The composition of fatty acids (FA) in the rumen is influenced by microorganisms and pH (Dewanckele et al., 2020). Saturated fatty acids (SFA) such as palmitic (C16:0) and stearic (C18:0), pass through the rumen unchanged. However, unsaturated fatty acids (UFA), including oleic (C18:1), linoleic (C18:2n-6), and linolenic (C18:3n-3), are chemically altered in the rumen, where microbes convert them into SFA to protect themselves from the potentially harmful effects of UFA (Lourenço et al., 2010). During this transformation, a variety of intermediate compounds are formed, including different types of conjugated linolenic acid (CLA) isomers (Lourenço et al., 2010). A decrease in the ruminal pH has been shown to inhibit the biohydrogenation process, ultimately suppressing the growth of CLA-producing bacteria. Grain-fed diets reduce lipolysis, which is a fundamental step in biohydrogenation (Lourenço et al., 2010). If ruminal biohydrogenation of UFA could be controlled, it might be possible to improve ruminant meat quality by increasing the deposition of UFA, CLA, and n-3 FA.

### Overall livestock health status in pastoral vs. intensive systems

Studies in grazing cattle, sheep, and goats generally report better animal welfare indicators, especially when ruminants can select a diverse mix of grasses, forages, and shrubs, allowing them to express their innate behaviors (Evans et al., 2024). Additional benefits of grazing are well-documented, including access to more space, fewer agonistic interactions, and better air quality (Falk et al., 2012).

Proper management of pastures containing different species can have a significant impact on animal welfare. A review by (Distel et al., 2020) examines the potential benefits of plant diversity for grazing ruminants. A diverse pasture could minimize disorders such as acidosis, hyperammonemia, bloating, and toxicity, which are more common in monoculture grazing. Associative effects between plants improve nitrogen retention and digestibility, which in turn reduces animal stress. Furthermore, the presence of condensed tannins or essential oils could contribute to reducing the carbon and nitrogen footprint. Although the authors also discuss some positive medicinal effects on animals, they remark that further research is needed to determine whether the combination of compounds in a diverse diet produces synergistic, antagonistic, or independent medicinal effects in grazing animals.

Oxidative stress, resulting from the imbalance between oxidizing and antioxidant compounds in favor of the oxidizing ones, has a key role in inflammation development and perpetuation in living beings (Falowo et al., 2014). The intake of plant antioxidants through grazing can improve the body’s oxidative status, strengthen the immune response, and reduce the apoptosis of intestinal epithelial cells, which promotes animal growth (Falowo et al., 2014). Among the antioxidant compounds present in fresh plants are vitamin E, vitamin C, carotenoid compounds, and polyphenols. Descalzo et al. (2005) observed that steers fed only fresh pastures reached plasma concentrations of antioxidants comparable with those of animals supplemented with vitamin E, suggesting that pastures can provide an equivalent proportion of antioxidant compounds.

Besides their oxidant action, polyphenols in the ruminant diet also exhibit anti-inflammatory properties and exert modulatory effects on metabolism and immunity (Vasta et al., 2019). In particular, condensed tannins have anthelmintic, antimicrobial, antiviral, and antidiarrheal properties, making them an alternative to veterinary drugs, helping to reduce the development of resistance. Santillo et al. (2022) demonstrated that condensed tannin intake helps mitigate oxidative stress caused by high temperatures in dairy cows and also helps prevent episodes of immune depression.

### Welfare and sustainability in pastoral and intensive systems

Although health and welfare are closely related, the first refers to physical and psychological well-being, while welfare is the relationship between the state of the animal and its environment, including management strategies as a part of it. In the United States, despite substantial improvements in cattle handling over the past 20 years, issues such as lameness, heat stress, heart failure, and liver abscesses in feedlot cattle have become common (Grandin, 2022). This author attributed these issues to genetic selection for increased weight gain, muddy pens, reduced forage in the ration, heavier cattle at a younger age, and the overuse of growth promoters, which may be detrimental to animal welfare.

Pastoral livestock finishing is often perceived as more ethical, healthier, and environmentally sustainable compared with confined systems (Evans et al., 2024). An Australian study surveyed consumer perceptions related to sheep and beef cattle welfare (Buddle et al., 2023). The participants perceived grazing systems as ‘natural-normal’ environments, in contrast to intensive production systems where animal movement and dietary choice are restricted. From a more anthropocentric point of view, pastoral ecosystems provide not only goods such as meat, milk, or fiber but also essential environmental services, including carbon sequestration and improved water infiltration capacity (Török et al., 2024).

Concerns from the industry about animal performance, profitability, and climate change must be seriously considered as critical factors for the feasibility of any sustainable production system. To answer both consumer and producer concerns, Klopatek et al. (2022) evaluated the environmental footprint (i.e., water use, land use, greenhouse gas emissions, and energy consumption), beef quality, and economic results of four systems: 1) grain-finished for 128 days, 2) grass-fed for 20 months, 3) grass-fed for 20 months followed by a 45-day grain finish, and 4) grass-fed for 25 months. The grain-finished steers exhibited the least greenhouse gas emissions but required the greatest energy input, while the 20 months grass-fed group used less water but produced the greatest greenhouse gas emissions. None of the systems showed superiority over the other in economic, meat quality, or environmental aspects.

## Food Safety, Meat Quality, and Human Health

Ensuring the safety and quality of animal products is essential for protecting human health. Consumers are increasingly concerned not only about the content of harmful substances, such as toxins or residues, but also about the nutritional value of the food they consume (Alonso et al., 2020). In this context, meat quality is influenced by various factors, including production systems and animal diets, which can affect both the safety of the product and its nutritional composition.

### Main hazards identified from the pastoral ecosystem to meat safety

The European Food Safety Authority (EFSA, 2013) identifies several biological and chemical hazards associated with beef consumption that may pose risks to human health. Biological hazards include pathogenic microorganisms such as Escherichia coli and Salmonella, which can cause severe foodborne illnesses. These pathogens are typically introduced through improper slaughtering procedures, leading to bacterial contamination of meat surfaces. Nevertheless, EFSA (2013) considers the biological risk from beef to be low to medium, probably because proper cooking, especially of ground meat, effectively eliminates these pathogens. Chemical hazards primarily involve contamination with toxic compounds such as dioxins and dioxin-like polychlorinated biphenyls. These substances are highly persistent in the environment and are primarily by-products of industrial activities, although they can also originate from natural sources like volcanic eruptions and forest fires, where combustion is incomplete. Dioxins accumulate on the surface of plants and soil and can be ingested by cattle when grazing contaminated pasture or inadvertently consuming soil. Once ingested, they accumulate in animal adipose tissue, resulting in higher concentrations in animals at the top of the food chain. Unlike pasture, grains do not typically carry dioxin residues, and intensive feeding systems tend to minimize animal contact with contaminated soil and groundwater (EFSA, 2013).

Dioxins are of significant concern due to their potential to disrupt hormonal function, damage the immune system, affect reproduction and development, and increase cancer risk. Although hundreds of dioxin-related compounds exist, only around 30 are known to cause considerable toxicity (WHO, 2019). According to the World Health Organization, more than 90% of human exposure to dioxins occurs through the consumption of animal products such as meat, dairy, and fish. While background levels of exposure are not considered an immediate health threat, the long-term risks underscore the need for strict monitoring of industrial emissions to prevent dioxin formation and accumulation along the food chain.

### Meat quality and human health

FA in both adipose tissue and muscle have a crucial role in determining the nutritional quality of meat. The main FAs found in meat include palmitic, stearic, oleic, linoleic, and linolenic acids. The last two are essential FA, which cannot be synthesized by the body and must be obtained through the diet (Wood et al., 2008).

Several studies indicate that consuming SFA raises LDL cholesterol, promotes inflammation, and increases cardiovascular disease risk, while monounsaturated FA (MUFA) and PUFA can counteract these effects. Also, Omega-6 (n-6) and omega-3 (n-3) FAs have cholesterol-reducing properties (Wood et al., 2008). WHO (2023) recommends diets with a PUFA:SFA ratio greater than 0.4 and limiting SFA intake to less than 10% of daily energy. However, an imbalanced n-6:n-3 ratio may cause adverse effects such as inflammation, thrombosis, hypertension, and neurological disorders (Daley et al., 2010). Thus, WHO (2023) also advises maintaining an n-6:n-3 ratio below 4.0. Additionally, linoleic and linolenic acids serve as precursors for long-chain (LC) PUFA synthesis, which are essential for cell membrane fluidity, the production of eicosanoids involved in platelet aggregation, thrombosis, inflammation, and gene expression (Wood et al., 2008).

The ruminant diet influences the meat proportion of SFA, MUFA, and PUFA, as well as the n-6:n-3 ratio. Increased body fat raises SFA and MUFA concentrations faster than PUFA, thus reducing PUFA:SFA ratio (Scollan et al., 2006). In contrast to grain-fed systems, animals in pastoral systems tend to be leaner, resulting in greater PUFA concentrations (Daley et al., 2010). Pasture-based diets can increase muscle linolenic acid and their LC-PUFA up to 4 times compared with grain-fed ruminants, significantly reducing the n-6:n-3 ratio in meat (Evans et al., 2024). Furthermore, the intramuscular fat (IMF) content and FA profile of meat from animals grazed on botanically diverse pastures may differ from those grazed on monophytic grasslands (Scollan et al., 2006). If the change in IMF and FA is qualitatively substantial, a favorable impact on human health can be expected. Subiabre et al. (2024) evaluated IMF and the FA profile of beef from cattle grazing a pasture of *Lolium perenne L*. plus *Bromus valdivianus*, and contrasted with a *Lolium multiflorum* pasture, showing higher intake values of FA of biomedical interest, and a higher IMF content. All pasture treatments showed n-6:n-3 favorable ratio. Other studies show that MUFAs are found in higher concentrations in the meat of grain-finished animals (Van Elswyk and McNeill, 2014), which can in turn be favorably influenced by animal genetics (Nogoy et al., 2022). Therefore, both systems (pasture-based and feedlot) could produce high-quality meat for human consumption.

A recent study with confined sheep found that α-linolenic acid in meat from hay-fed animals was 71% to 108% higher than in grain-fed groups (Villaverde, 2025; Table 2). However, no significant differences were observed in SFA, UFA, PUFA concentrations, or the PUFA:SFA ratio, which remained below the minimum ideal value. The n-6:n-3 ratio was lower in hay-fed sheep than in grain-fed animals but remained above the ideal threshold in all diets.

**Table 2.** Composition of fatty acid content in the meat of sheep fed hay-based (hay), oat-based (oat), or corn-based (corn) diets^*^

Fatty acids in meat, % of total	Diet^†^			*P*-value^‡^
	Hay	Oat	Corn	
Palmitic (C16:0)	23.6	22.7	24.0	NS
Stearic (C18:0)	17.2	20.4	16.6	NS
Oleic (C18:1)	31.1	33.4	36.8	0.04
Linoleic (C18:2n-6)	5.5	5.9	5.5	NS
α-linolenic (C18:3n-3)	1.2	0.6	0.7	0.03

^*^ Villaverde, 2025.

^†^Hay: 99 % chopped pasture hay from a mix of perennial grasses and 1% sunflower meal; Oat: 60% of whole oat grain, 32% chopped pasture hay, and 8 % sunflower meal; Corn: 60% whole corn grain, 30% of chopped pasture hay, and 10% sunflower meal. Diets were formulated to provide equal protein content and were offered ad libitum in individual stalls.

^‡^NS: non-significant differences, *P *> 0.05.

The conversion of UFA to SFA by ruminal biohydrogenation represents a major human health concern. Pasture feeding stimulates saliva production, which sustains a higher rumen pH that favors lipolysis and biohydrogenation (Lourenço et al., 2010). In grazing cattle, trans-vaccenic acid (TVA, C18:1 t11) is a major biohydrogenation intermediate in the rumen and a precursor of CLA at the tissue level. The 18:2 cis-9, trans-11 isomer accounts for 80% of CLA produced. Conjugated linoleic acid isomers are linked to health benefits, including protection against coronary heart disease, obesity, cancer, atherosclerosis, and diabetes (Wood et al., 2008). Pasture-fed ruminants produce two to three times more CLA than those on high-concentrate diets, making pasture beef and other ruminant products important dietary sources of CLA (Daley et al., 2010). A study with steers grazing on oat pasture evaluated the effect of daily supplementation with different amounts of whole oat grain on the meat lipid profile (Arelovich et al., 2016). Results showed that oats supplementation with up to 0.50% of body weight had little impact on the fatty acid profile, with no differences in CLA concentration among treatments (Table 3). Additionally, the n-6: n-3 ratio was lower in steers fed no grain, although all treatments remained below the maximum recommended value by the WHO.

**Table 3.** Fatty acid composition of beef from cattle fed oat pasture or oat pasture supplemented with oat grain at 0.25% or 0.50% of body weight ^*^

Fatty acids in meat, % of total	Diet			*P*-value^†^
	Pasture	0.25% Oat	0.50% Oat	
Palmitic (C16:0)	23.73	24.13	25.12	NS
Stearic (C18:0)	13.38	13.27	13.35	NS
Trans-vaccenic (C18:1 t11)	1.70	1.38	1.37	0.05
Oleic (C18:1)	29.47	31.15	31.00	NS
Linoleic (C18:2n-6)	2.88	3.23	2.83	0.05
α-linolenic (C18:3n-3)	1.12	1.05	0.75	0.01
CLA (C18:2 c9-t11)	0.45	0.40	0.38	NS

^*^ Arelovich et al., 2016.

^†^NS: non-significant differences, *P* > 0.05.

Grazing also favors the accumulation of phytochemicals (polyphenols, terpenes, carotenoids, and other antioxidant compounds) in meat, which are directly related to the concentration of these compounds in the forages consumed (Evans et al., 2024). Condensed tannin consumption can also reduce or modulate the biohydrogenation of PUFA by altering the composition and microbial activity in the rumen; thus, promoting greater incorporation of these FAs into the meat (Vasta et al., 2019).

A recent meta-analysis on the impact of meat consumption on human cardiovascular health criticizes the generalization of outcomes across all red meats, despite species differences (beef, sheep, goat, and pork; Sanders et al., 2024). The authors indicate that red meats vary in composition and nutritional value, and not all studies show a direct association between red meat intake and cardiovascular disease. They conclude that frequent beef consumption provides highly bioavailable protein, iron, zinc, and selenium without significantly affecting lipid profiles or blood pressure compared to lower beef intake, white meat, or meat-free diets (Sanders et al., 2024).

## Final Considerations on OH Interactions: Pastoral Systems-Animal-Human

Grazing ruminants are an important source of meat and milk, both of which are highly valued for their nutritional and economic benefits. Successful production in pasture-based systems depends heavily on factors such as rainfall, forage availability and quality, and pasture type. When managed well, pastoral ecosystems enhance soil health through improved nutrient cycling, erosion prevention, and carbon sequestration, which in turn supports water runoff prevention and biodiversity of plants and wildlife.

Although intensive or confined systems are more efficient in feed utilization, weight gain, and final body weight, they often compromise animal welfare and contribute to environmental pollution. As discussed above, pasture-fed systems produce leaner meat rich in n-3 FAs, CLA, and vitamins A and E, while containing less total fat and SFA. In contrast, grain-based systems tend to produce meat with a higher concentration of MUFA than pasture-based systems. However, compared to grain finishing, pasture-based systems fit more naturally within the OH paradigm, due to their lower environmental impact and potential benefits for animal welfare. Additionally, cost-effectiveness must be considered by recognizing the economic value and benefits of properly managed pastoral systems, along with their contribution to a more ethical approach to meat production, which benefits human health and enhances consumer satisfaction.

## Author Biography

**María Sol Villaverde** is an Agricultural Engineer and holds a PhD in Agronomy from the Universidad Nacional del Sur (UNS, Argentina), where she conducted her doctoral research under a National Scientific and Technical Research Council (CONICET) fellowship. She serves as a Teaching Assistant in the Beef and Sheep Production course at Department of Agronomy (UNS) and is a member of the Animal Nutrition Laboratory and the Laboratory of Natural and Agricultural Ecosystems of the Commission for Scientific Research of the Province of Buenos Aires (CIC-PBA). Her research focuses on animal nutrition and meat quality in ruminants.

**Mariano Menghini** is an Agricultural Engineer and holds a PhD in Agronomy from the Universidad Nacional del Sur (Argentina). He is currently an Assistant Professor with primary teaching responsibilities in the courses of Animal Physiology and Nutrition, and Ruminant Nutrition. His international experience includes research internships at Oklahoma State University (USA), Universidad de Córdoba (Spain), and Universidade Federal Rural do Rio de Janeiro (Brazil). His main research interests are grazing animal nutrition and pasture production and quality.

**Marcela F. Martínez** is a Biochemist and holds a PhD in Agronomy from the Universidad Nacional del Sur (Argentina), where she is currently an Associate Professor in the Department of Agronomy. She teaches Animal Science-related courses and is responsible for the Animal Nutrition Laboratory. Her research focuses primarily on ruminant nutrition, with emphasis on the use of low-quality roughages, as well as oat grain and forage, and their implications for meat quality. She has also contributed to non-ruminant research and held administrative positions, including serving as Vice Director of the Department.

**Nicolás DiLorenzo** is an Agricultural Engineer from the Universidad Nacional de La Plata (Argentina). He earned his MSc and PhD degrees in Animal Science, specializing in beef cattle nutrition, from the University of Minnesota (USA). From 2008 to 2010, he was a Postdoctoral Research Associate at Texas Tech University (USA), conducting research on feedlot nutrition and management. In 2010, he joined the University of Florida as an Assistant Professor of Animal Sciences at the North Florida Research and Education Center in Marianna. His research and extension programs focus on improving forage utilization efficiency while reducing environmental impact, with emphasis on ruminal metabolism and fermentation, greenhouse gas emissions, and nutrient excretion in cattle systems.

**Rodrigo D. Bravo** is an Agricultural Engineer and holds an MSc in Agronomy from the Universidad Nacional del Sur (Argentina), where he is an Assistant Professor of Animal Nutrition in the Department of Agronomy. He teaches both undergraduate and graduate courses and coordinates academic activities within the Department. He also serves as Director of the university’s experimental fields, where he has promoted the expansion of field-based teaching, research, and outreach to rural communities through extension activities and strategic partnerships with public and private institutions.

**Hugo M. Arelovich** is an Agricultural Engineer from the Universidad Nacional del Sur (Argentina) and holds MSc and PhD degrees from Oklahoma State University (USA). After 39 years as a faculty member at the Universidad Nacional del Sur, he was awarded the title of “Profesor Extraordinario Consulto,” allowing him to remain academically active. He has taught undergraduate and graduate courses, conducted research in ruminant nutrition, and served two nonconsecutive terms as President of the Argentine Association of Animal Production (AAPA). He has been involved in numerous professional, academic, and producer-oriented activities in Argentina and abroad, and has received distinctions from Oklahoma State University and the American Society of Animal Science. He currently works as a freelance consultant in animal science.
